# Preclinical Evaluation of 5F-αMe-3BPA for Improving Pharmacokinetics in Boron Neutron Capture Therapy

**DOI:** 10.3390/pharmaceutics18050604

**Published:** 2026-05-15

**Authors:** Naoya Kondo, Fuko Hirano, Saki Iritani, Kensuke Suzuki, Anna Miyazaki, Takashi Temma

**Affiliations:** 1 Department of Biofunctional Analysis, Graduate School of Pharmaceutical Sciences, Osaka Medical and Pharmaceutical University, 4-20-1 Nasahara, Takatsuki 569-1094, Osaka, Japan; kondo.ny@kmu.ac.jp (N.K.); fuhiran@east.ncc.go.jp (F.H.); anna.miyazaki@ompu.ac.jp (A.M.); 2 Division of Fundamental Technology Development, Near InfraRed Photo-ImmunoTherapy Research Institute, Kansai Medical University, 2-5-1 Shin-machi, Hirakata 573-1010, Osaka, Japan; 3 Stella Pharma Corporation Sakai R&D Center, Bldg. C-23, 1-1 Gakuen-cho, Naka-ku, Sakai 599-8531, Osaka, Japan; ksuzuki@stella-pharma.co.jp; 4 Laboratory of Quantum Biological Analysis, Institute for Integrated Radiation and Nuclear Science, Kyoto University, 2-1010 Asashiro Nishi, Kumatori, Sennan-gun 590-0494, Osaka, Japan

**Keywords:** boron neutron capture therapy (BNCT), L-type amino acid transporter 1 (LAT1), borono-phenylalanine (BPA), probenecid, organic anion transporter 1 (OAT1)

## Abstract

**Background/Objectives**: Boron neutron capture therapy (BNCT) relies on the selective delivery of boron-10 to tumor cells. Although 4-[^10^B]borono-L-phenylalanine (BPA) is currently the only clinically approved BNCT agent, it is limited by poor L-type amino acid transporter 1 (LAT1)/LAT2 selectivity and aqueous solubility. We previously developed 3-borono-5-fluoro-α-methyl-L-phenylalanine (5F-αMe-3BPA), a novel BPA derivative designed to be a LAT1-targeted BNCT/positron emission tomography theranostic agent. This study comprehensively characterizes its pharmacological profile and explores its pharmacokinetic optimization by modulating renal organic anion transporter 1 (OAT1). **Methods**: Transport kinetics of BPA, related analogs, and 5F-αMe-3BPA were analyzed in HEK293 cells stably expressing LAT1 or LAT2 using Michaelis–Menten analysis. Time-dependent cellular uptake and intracellular retention of BPA and 5F-αMe-3BPA were evaluated in T3M-4 pancreatic cancer cells with or without the LAT1 inhibitor JPH203. In vivo biodistribution was examined in T3M-4 tumor-bearing mice after intravenous administration of 5F-αMe-3BPA or BPA, with assessment of probenecid pretreatment. **Results**: 5F-αMe-3BPA retained LAT1 affinity comparable to that of BPA while showing markedly reduced LAT2-mediated transport, indicating improved LAT1/LAT2 selectivity. In T3M-4 cells, 5F-αMe-3BPA showed stronger LAT1 dependence, higher steady-state accumulation, and better intracellular retention than BPA under amino acid-containing conditions. Although 5F-αMe-3BPA achieved favorable tumor-to-plasma and tumor-to-muscle ratios in vivo, it was rapidly cleared from circulation. Probenecid pretreatment increased plasma exposure, reduced early renal accumulation, and significantly enhanced tumor boron accumulation, reaching approximately twofold higher levels than control. **Conclusions**: These findings establish 5F-αMe-3BPA as a highly LAT1-selective BNCT candidate and identify probenecid pretreatment as a clinically translatable pharmacokinetic strategy for maximizing therapeutic boron delivery.

## 1. Introduction

Boron neutron capture therapy (BNCT) is a targeted radiotherapy that exploits the nuclear reaction between boron-10 (^10^B) and thermal neutrons to generate high linear energy transfer (LET) particles—specifically, alpha particles (^4^He) and lithium-7 (^7^Li) nuclei—with an extremely short range of <1 cell diameter (~10 μm) [1,2]. When ^10^B is selectively localized to cancer cells, BNCT theoretically achieves tumor-selective cytotoxicity while sparing the surrounding normal tissues, providing a unique approach to precision radiotherapy [3]. Currently, 4-[^10^B]borono-L-phenylalanine (BPA), marketed as borofalan in Japan, remains the only clinically approved BNCT agent for treating unresectable, locally advanced, or locally recurrent head and neck cancers [4,5]. BPA is primarily transported to tumor cells by L-type amino acid transporter 1 (LAT1) [6,7], which is overexpressed in various cancers [8]. Despite its clinical utility, BPA-based BNCT has two major limitations. First, BPA has extremely low aqueous solubility (0.7 g/L), and the dose required for therapy (500 mg/kg) cannot be formulated using BPA alone. As a result, BPA must be administered as a sugar complex to improve its solubility [9,10]. Second, BPA demonstrates low transporter subtype specificity, functioning as a substrate for LAT1 and LAT2 [7]. In tumors wherein LAT1 expression predominates, cellular transport occurs primarily via LAT1. However, in normal tissues that express LAT2 but lack LAT1, BPA uptake is mediated by LAT2 [7,11]. Consequently, BPA often yields modest tumor-to-normal tissue boron concentration ratios (~4) [12,13], compelling clinicians to limit neutron fluence, and therefore tumor dose, to remain within normal tissue tolerance, thereby narrowing the therapeutic window [14].

To overcome these limitations, we previously conducted a series of structure–property optimizations starting from BPA. Relocation of the boronic acid moiety from the para position to the meta position yielded 3-borono-L-phenylalanine (3BPA), which exhibits more than 100-fold higher aqueous solubility while maintaining comparable tumor-targetability to BPA [15]. Subsequent α-methylation of 3BPA (αMe-3BPA) substantially reduced LAT2 transport and increased LAT1/LAT2 selectivity, enhancing tumor-to-normal tissue boron ratios in a LAT1-expressing xenograft mouse model (~11.0) [16]. Building on this scaffold, 5F-αMe-3BPA, a fluorinated derivative with a fluorine atom introduced at position 5 of the phenyl ring of αMe-3BPA, further improved physicochemical properties and enabled straightforward ^18^F-labeling for positron emission tomography (PET) using an identical molecular structure, positioning it as a BNCT/PET theranostic candidate [17].

Initial screening of 5F-αMe-3BPA demonstrated LAT1-dependent uptake and high tumor-to-normal tissue ratios. However, in vitro and in vivo evaluations were limited to a single time point, and the temporal dynamics of cellular uptake and efflux, as well as the in vivo pharmacokinetics, remain insufficiently characterized. Notably, low plasma boron concentration and high renal accumulation suggested rapid kidney-mediated clearance of 5F-αMe-3BPA, potentially via organic anion transporter 1 (OAT1)-mediated tubular secretion, as reported for other halogenated and α-methylated aromatic amino acids [18,19]. These observations suggest that pharmacokinetic modulation strategies that prolong systemic exposure of 5F-αMe-3BPA without compromising its intrinsic LAT1 selectivity might further improve boron delivery to LAT1-expressing tumors, enhancing BNCT efficacy.

Therefore, the present study sought to (i) quantitatively characterize the LAT1 and LAT2 transport kinetics of BPA, 5F-αMe-3BPA, and their developmental intermediates; (ii) compare the time-dependent uptake and intracellular retention of BPA and 5F-αMe-3BPA in LAT1-expressing cancer cells; and (iii) evaluate probenecid, a clinical OAT1 inhibitor, as a pharmacokinetic modulator to determine whether OAT1 inhibition improves tumor accumulation of 5F-αMe-3BPA in vivo. Collectively, these analyses provide critical evidence supporting 5F-αMe-3BPA as a highly LAT1-selective BNCT/PET theranostic candidate and establish probenecid-mediated OAT1 inhibition as a clinically translatable strategy to enhance tumor boron delivery.

## 2. Materials and Methods

### 2.1. General

All reagents were purchased from Fujifilm Wako Pure Chemical Corporation (Osaka, Japan) or Nacalai Tesque (Kyoto, Japan) and were used as received. ^10^B-enriched BPA was provided by Stella Pharma Corporation (Osaka, Japan). 3BPA, αMe-BPA, αMe-3BPA, and 5F-αMe-3BPA were synthesized according to previously reported methods [16] and were supplied by Stella Pharma Corporation.

### 2.2. Cell Culture

Human pancreatic adenocarcinoma T3M-4 cells were provided by the RIKEN BioResource Research Center (Ibaraki, Japan) and were maintained in RPMI 1640 medium supplemented with 10% fetal bovine serum and 1% penicillin-streptomycin at 37 °C in a humidified atmosphere with 5% CO_2_. HEK293 human embryonic kidney cell lines stably expressing human LAT1 (HEK-LAT1) and LAT2 (HEK-LAT2) were generated and cultured following previously reported protocols [20]. The established cell lines were provided by Stella Pharma Corporation.

### 2.3. Michaelis–Menten Kinetic Analysis

HEK-LAT1 and HEK-LAT2 cells were seeded in type I collagen-coated 12-well plates three days before the experiments. After removing the culture medium, the cells were washed twice with Na^+^-free Hank’s balanced salt solution (HBSS; 125 mM choline chloride, 25 mM HEPES, 4.8 mM potassium chloride, 5.6 mM D-glucose, 1.3 mM calcium chloride, 1.2 mM magnesium sulfate, and 1.2 mM potassium dihydrogen phosphate; pH 7.4) and preincubated with 200 µL HBSS at 37 °C for 10 min. Following preincubation, 100 μL of the compounds at various concentrations (final concentrations: 7.8, 15.6, 31.3, 62.5, 125, 250, 500, and 1000 µM) were added, and the cells were incubated at 37 °C for 10 min. The uptake reaction was terminated by washing twice with ice-cold HBSS, and the cells were lysed with 300 µL of 0.5% Tween 20. The boron content was determined fluorometrically (λex/λem: 355/460 nm) using a plate reader (EnSpire Multilabel Reader 2300, PerkinElmer Japan, Kanagawa, Japan) after the addition of 10 mM 2-(2-hydroxyphenyl)pyridine [21] followed by incubation for 30 min at 37 °C. The boron concentration was determined using a calibration curve, and the number of cells per well was counted. Uptake velocities (V, fmol·cell^−1^·min^−1^) at each substrate concentration were plotted, and the Michaelis constant (Km) was calculated with nonlinear regression analysis using GraphPad Prism 8 software based on the Michaelis–Menten equation (*n* = 3, performed in triplicate).

### 2.4. Time-Course Cellular Uptake Assay

T3M-4 cells were cultured in 12-well dishes. After removing the medium, the cells were washed twice with HBSS and preincubated with 450 µL of HBSS at 37 °C for 5 min. Uptake was initiated by adding 50 μL of 1 mM 5F-αMe-3BPA or BPA–fructose complex (final concentration: 100 µM) and incubating at 37 °C for 10, 30, 60, 120, or 240 min. After removing the solution and washing twice with HBSS, 400 μL of 0.2 M NaOH was added to lyse the cells. For the LAT1 inhibition analyses, the cells were preincubated with HBSS containing 10 µM JPH203, a LAT1 inhibitor, at 37 °C for 5 min using 450 μL of the solution. The cellular protein content in each well was measured. Cell lysates were washed using nitric acid at 120 °C for 3 h, and the boron content was determined using 8800 triple quadrupole inductively coupled plasma mass spectrometry (ICP-MS, Agilent, Santa Clara, CA, USA). Boron accumulation was calculated as the percentage of the injected dose per milligram (%ID/mg) protein (*n* = 5, performed in triplicate).

### 2.5. Intracellular Retention Assay

T3M-4 cells were incubated with each compound for 60 min using the method described in Section 2.4. After removing the solution and washing twice with HBSS, 500 μL of HBSS or RPMI 1640 medium was added, and the cells were incubated for 5, 10, or 30 min. After removing the solution, cellular protein and boron content were measured as described in Section 2.4. Boron retention was calculated as the percentage of the initial loaded amount (60-min time point set to 100%) and normalized to protein content (*n* = 3, performed in triplicate).

### 2.6. Animal Studies

Male BALB/cSlc-nu/nu mice (*n* = 31, 4 weeks old) were obtained from Japan SLC Inc. (Shizuoka, Japan) and maintained under a 12-h light/dark cycle with ad libitum access to food and water. For tumor inoculation, T3M-4 cells (2.5 × 10^6^) suspended in 100 μL of medium were subcutaneously injected into the right hind leg under isoflurane anesthesia to minimize discomfort. Mice were randomized into experimental groups based on tumor size (>100 mm^3^) to minimize intergroup variability. All animal experiments were performed in accordance with institutional guidelines and were approved by the institutional animal care and use committee (Permission Number: AP23-004). Tumors exceeding 1000 mm^3^ or exhibiting central necrosis at the time of experimentation were excluded from the analysis. To ensure unbiased results, the experiments were conducted in a blinded manner. Specifically, drug administration and boron measurements were performed by separate investigators to minimize potential bias.

### 2.7. In Vivo Biodistribution Studies

Tumor-bearing mice (8–9 weeks old, 21–25 g) received 100 μL of 50 mM 5F-αMe-3BPA or BPA-fructose complex via tail vein injection. At 10 min (*n* = 6 for 5F-αMe-3BPA) and 180 min (*n* = 6 for 5F-αMe-3BPA, *n* = 4 for BPA) post-injection, the mice were euthanized, and tumors along with selected organs were harvested and weighed. The samples were washed, and the boron concentrations were determined using ICP-MS. Endogenous boron levels in tissues from untreated mice were subtracted as background. Boron accumulation in each tissue was expressed as %ID/g.

For the probenecid-treated group, probenecid was dissolved in phosphate-buffered saline (PBS, pH 7.4) at a concentration of 50 mM and administered intraperitoneally at a dose of 400 mg/kg, 60 min before tail vein injection of 5F-αMe-3BPA. At 10, 60, and 180 min post-injection, mice were euthanized and tissues were harvested as described above (*n* = 5 per time point). The area under the %ID/g–time curve (%ID/g·min, AUC_10–60_, AUC_60–180_, and AUC_10–180_) for each tissue was estimated at three time points using the linear trapezoidal method. For the analysis of biodistribution studies, part of the control data at 60 min post-injection were reproduced from our previous study [17], which were obtained under the same experimental conditions.

### 2.8. Statistical Analysis

All data were presented as mean ± standard deviation. Statistical analyses were performed using the GraphPad Prism 8 software. Comparisons between two groups were performed using an unpaired Student’s *t*-test, and multiple group comparisons were performed using one-way analysis of variance, followed by Dunnett’s multiple comparisons test. The specific statistical tests used are indicated in the corresponding figure legends and table footnotes. A *p*-value of <0.05 was considered statistically significant.

## 3. Results

### 3.1. Michaelis–Menten Kinetic Analysis of LAT1 and LAT2 Transport

**Figure 1:**
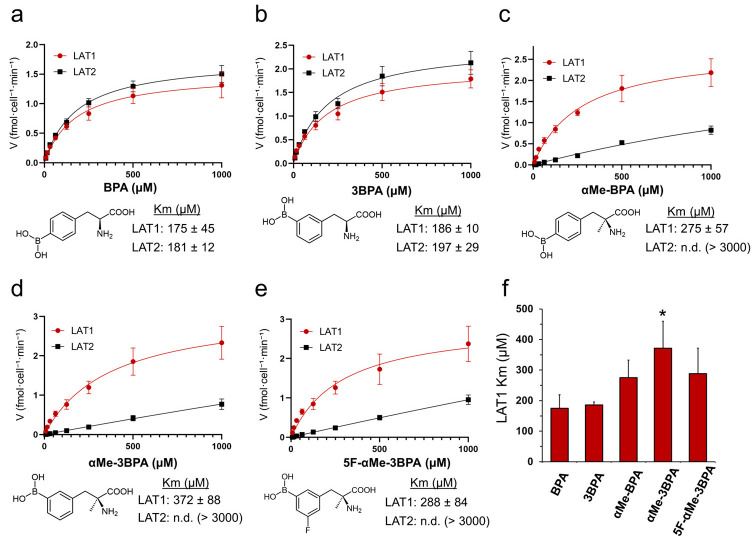
Transport kinetics of boronophenylalanine derivatives via LAT1 and LAT2. Michaelis–Menten plots showing the uptake of (**a**) BPA, (**b**) 3BPA, (**c**) αMe-BPA, (**d**) αMe-3BPA, and (**e**) 5F-αMe-3BPA in HEK293 cells stably expressing human LAT1 or LAT2. (**f**) Comparison of Km values for LAT1-mediated transport. Data represent mean ± SD (*n* = 3, performed in triplicate). * *p* < 0.05 versus BPA (Dunnett’s multiple comparisons test). n.d., could not be determined due to poor model fitting.

Michaelis–Menten plots and derived Km values for BPA, 3BPA, αMe-BPA, αMe-3BPA, and 5F-αMe-3BPA in HEK293 cells stably expressing LAT1 or LAT2 are shown in Figure 1a–e. For LAT1-mediated transport, 3BPA exhibited an affinity comparable to that of BPA (Km = 186 µM vs. 175 µM; *p* = 0.99). Among the α-methylated derivatives, αMe-3BPA demonstrated a modest but statistically significant decrease in LAT1 affinity (Km = 372 µM, *p* = 0.01 vs. BPA), whereas αMe-BPA (Km = 275 µM, *p* = 0.22) and 5F-αMe-3BPA (Km = 288 µM, *p* = 0.15) were not significantly different from BPA (Figure 1f).

Conversely, for LAT2-mediated transport, 3BPA had an affinity comparable to that of BPA (Km = 197 µM vs. 181 µM, *p* = 0.99) (Figure 1a,b). Notably, all α-methylated derivatives showed substantially reduced LAT2 affinity, with Km values exceeding 3000 µM, which could not be reliably determined due to poor fitting of the Michaelis–Menten equation. This finding indicated a substantial loss of LAT2-mediated transport (Figure 1c–e).

### 3.2. Time-Dependent Cellular Uptake and Intracellular Retention

**Figure 2:**
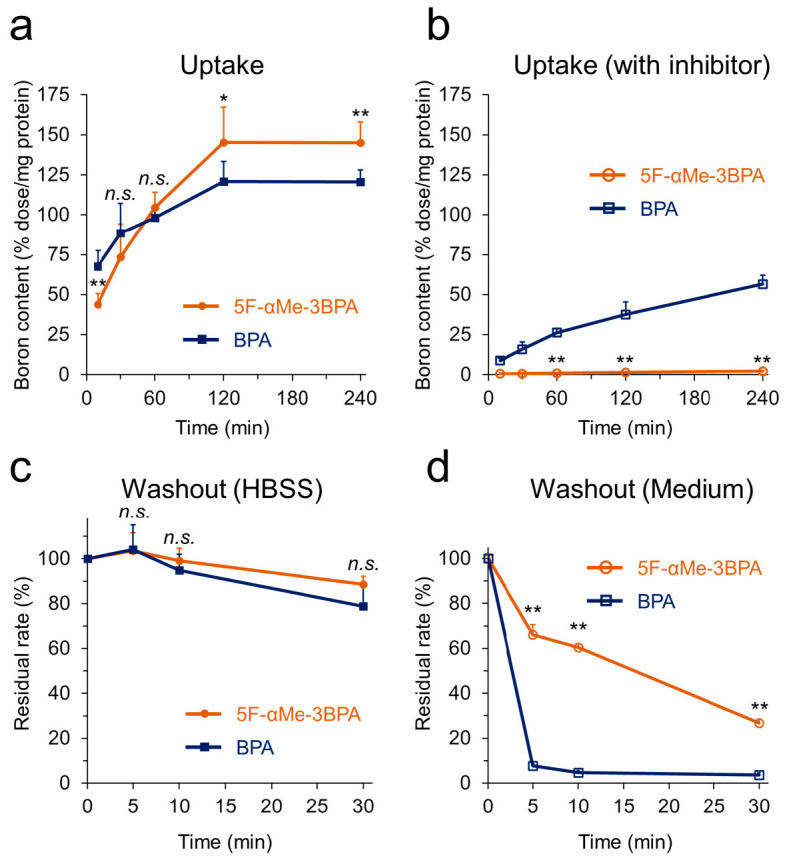
Time course of cellular uptake and intracellular retention of BPA and 5F-αMe-3BPA in T3M-4 cells. T3M-4 cells were incubated with 100 µM BPA–fructose complex or 5F-αMe-3BPA at 37 °C. (**a**,**b**) Time-dependent cellular boron accumulation at 10–240 min with (**b**) or without (**a**) JPH203 (10 µM), a selective LAT1 inhibitor. (**c**,**d**) Intracellular retention of boron after a 60-min loading period followed by incubation in amino acid-free HBSS (**c**) or amino acid-containing RPMI 1640 medium (**d**) for the indicated times. Values were normalized to the 0 min time point (set to 100%). Boron content was determined using ICP-MS and normalized to protein content. Data represent mean ± SD (*n* = 5 for (**a**,**b**); *n* = 3 for (**c**,**d**); each performed in triplicate). * *p* < 0.05, ** *p* < 0.01 n.s., not significant, versus BPA at the same time point (unpaired *t*-test).

The time course of boron accumulation in T3M-4 cells following the addition of BPA and 5F-αMe-3BPA is shown in Figure 2a. Boron content in cells 10 min after incubation with 5F-αMe-3BPA (44% ± 7% dose/mg protein) was significantly lower than that after BPA incubation (68% ± 10% dose/mg protein), whereas the accumulation of the two compounds was comparable at 30 min. At 120 min, 5F-αMe-3BPA accumulation (145% ± 22% dose/mg protein) was significantly higher than BPA accumulation (121% ± 13% dose/mg protein), which plateaued thereafter.

In the presence of JPH203, a selective LAT1 inhibitor, accumulation was significantly lower at all time points (Figure 2b). Under JPH203 co-incubation, a minimal uptake of 5F-αMe-3BPA was observed (2.1% ± 0.6% dose/mg protein) even at 240 min, whereas BPA accumulated progressively, reaching 57% ± 5% dose/mg protein at 240 min and demonstrating significantly higher accumulation compared to 5F-αMe-3BPA from 60 min onward.

Intracellular retention assays revealed no significant differences between 5F-αMe-3BPA and BPA levels in amino acid-free HBSS at any time point (89% ± 4% vs. 79% ± 8% retention at 30 min for 5F-αMe-3BPA and BPA, respectively; Figure 2c). In contrast, with amino acid-containing RPMI 1640 medium, a clear difference emerged from 5 min onward: 5F-αMe-3BPA retained 66% ± 4% of intracellular boron, whereas BPA retained only 8% ± 2%, indicating rapid efflux of BPA (Figure 2d). At 30 min, 5F-αMe-3BPA retained 27% ± 2% of intracellular boron, whereas BPA retained 4% ± 1%.

### 3.3. Pharmacokinetic Comparison of BPA and 5F-αMe-3BPA

**Figure 3:**
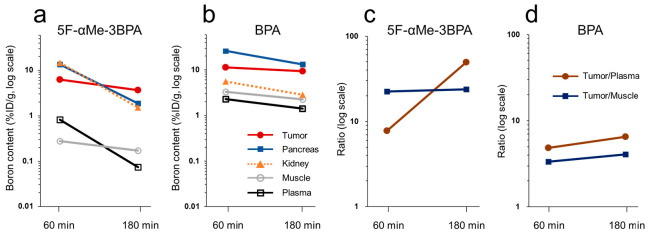
Time-dependent changes in boron accumulation after the administration of 5F-αMe-3BPA or BPA in T3M-4 tumor-bearing mice. (**a**,**b**) Time-dependent changes in boron accumulation in T3M-4 tumor, pancreas, kidney, muscle and plasma at 60 and 180 min after administration of (**a**) 5F-αMe-3BPA and (**b**) BPA. (**c**,**d**) Time-dependent changes in tumor-to-plasma and tumor-to-muscle boron concentration ratios at 60 and 180 min after the administration of (**c**) 5F-αMe-3BPA and (**d**) BPA. The 60-min data for both 5F-αMe-3BPA and BPA were reproduced from our previous study [17], while the 180-min data were obtained in the present study.

Boron pharmacokinetics after intravenous administration of 5F-αMe-3BPA or BPA in T3M-4 tumor-bearing mice is shown in Figure 3, with the accumulated boron content shown on a logarithmic scale. 5F-αMe-3BPA displayed rapid and parallel boron washout from the plasma and kidney to approximately one-tenth of the initial value over the time period, whereas tumors showed a slow decrease, remaining at 60% of the initial value (Figure 3a). This phenomenon contributed to a progressive increase in the tumor-to-plasma ratio, which reached up to 49 (Figure 3c). Accumulation of 5F-αMe-3BPA in normal tissues was generally low, and the tumor-to-muscle ratio remained consistently higher than 20. Conversely, BPA was slowly cleared from all organs, retaining more than 50–70% of the initial values until 180 min (Figure 3b). Therefore, the tumor-to-plasma ratio increased only modestly, reaching 6.6 (Figure 3d).

**Figure 4:**
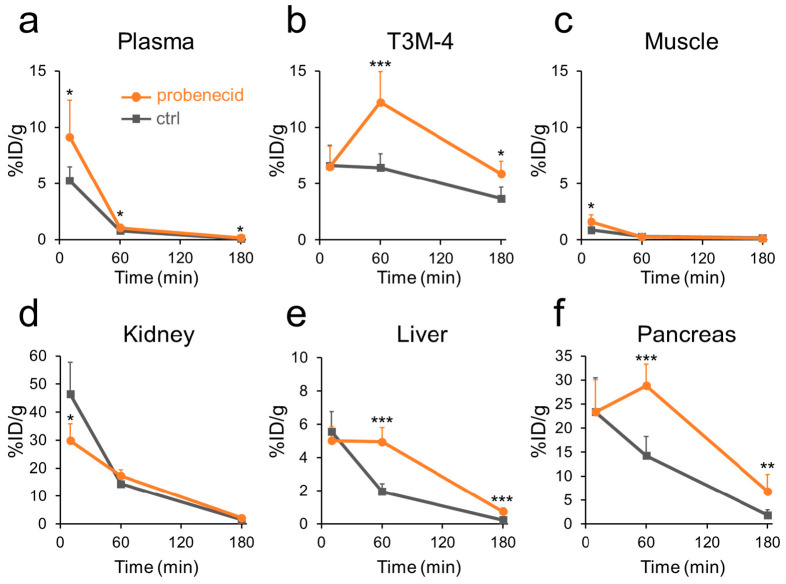
Effect of probenecid on the 5F-αMe-3BPA pharmacokinetics in T3M-4 tumor-bearing mice. Time course of boron accumulation in (**a**) plasma, (**b**) T3M-4 tumor, (**c**) muscle, (**d**) kidney, (**e**) liver, and (**f**) pancreas tissue following intravenous administration of 5F-αMe-3BPA with (circles) or without (squares) probenecid pretreatment. Endogenous boron levels in untreated mice were subtracted as the background. Data are expressed as %ID/g (mean ± SD). * *p* < 0.05, ** *p* < 0.01, *** *p* < 0.001 versus the control group at the same time point (unpaired *t*-test). The 60-min control data were reproduced from our previous study [17], while the others were obtained in the present study. The 180-min control data are the same as those presented in Figure 3.

To assess pharmacokinetic modulation, the effect of probenecid pretreatment on boron pharmacokinetics after intravenous administration of 5F-αMe-3BPA was evaluated. The boron content was measured at three time points, including an earlier time point at 10 min (Figure 4). Detailed boron accumulation data are provided in Table S1, and the AUC values calculated at the observed time points are shown in Table S2. Probenecid pretreatment significantly reduced kidney accumulation at 10 min (30%ID/g vs. 46%ID/g, *p* < 0.05, Figure 4d) and increased plasma boron levels at all evaluated time points, especially at 10 min (Figure 4a). Thus, probenecid led to significantly higher tumor boron accumulations at both 60 (12%ID/g vs. 6.2%ID/g, *p* < 0.001) and 180 min (5.9%ID/g vs. 3.7%ID/g, *p* < 0.05) (Figure 4b), accompanied by higher tumor-to-muscle ratios. The AUC data also demonstrated increased tumor boron accumulation and a higher tumor-to-muscle ratio in the probenecid group (AUC_10–180_, %ID/g·min, tumor_ctrl_ = 922, tumor_probenecid_ = 1557, muscle_probenecid_ = 73). Boron accumulation in the pancreas, representative of LAT1-expressing normal tissues [22], was also significantly increased by probenecid at 60 (29%ID/g vs. 13%ID/g, *p* < 0.001) and 180 min (6.8%ID/g vs. 1.9%ID/g, *p* < 0.01) (Figure 4f). Additionally, liver boron concentrations were significantly elevated in the probenecid group starting at 60 min (Figure 4e).

## 4. Discussion

This study provides a comprehensive pharmacological characterization of 5F-αMe-3BPA and demonstrates that probenecid-mediated renal OAT inhibition enhances tumor delivery while largely preserving tumor selectivity. 5F-αMe-3BPA was developed through stepwise structural optimization of BPA, including meta-isomerization to improve aqueous solubility (3BPA) [15], α-methylation to increase LAT1/LAT2 selectivity (αMe-3BPA) [16], and fluorination to enable structurally matched PET imaging and further refine physicochemical properties (5F-αMe-3BPA) [16,17]. To quantitatively assess transporter selectivity, we measured the Km values in HEK293 cells stably expressing LAT1 or LAT2 for five compounds (BPA, 3BPA, αMe-BPA, αMe-3BPA, and 5F-αMe-3BPA). The kinetic analyses clearly identified α-methylation as the key structural determinant that markedly improves LAT1/LAT2 selectivity by abolishing LAT2-mediated transport, while preserving LAT1 affinity. Conversely, the positional isomerism of the boronic acid moiety (para vs. meta) had a minimal impact on transporter affinity, and fluorination did not compromise LAT1 recognition. Together, these findings suggest that the superior selectivity of 5F-αMe-3BPA primarily arises from α-methylation. Although the absolute Km values obtained in the present study are distinct from those reported in Xenopus oocyte expression systems [7], these differences likely arise from variations in the experimental platforms, species orthologs, and assay conditions. Nevertheless, within-study comparison across five analogs provides robust support for a clear structure–selectivity relationship and highlights α-methylation as the primary determinant of LAT1/LAT2 selectivity in this chemical series.

Time-course cellular uptake experiments in T3M-4 pancreatic cancer cells revealed that 5F-αMe-3BPA exhibits slower initial uptake but achieves higher steady-state accumulation than BPA. This reduced initial uptake likely reflects the lower LAT1 affinity of 5F-αMe-3BPA relative to BPA, although this difference did not reach statistical significance. More importantly, the superior steady-state accumulation of 5F-αMe-3BPA implies reduced efflux relative to BPA, leading to higher intracellular boron levels at equilibrium. The near complete abrogation of 5F-αMe-3BPA uptake by the LAT1 inhibitor JPH203 confirms its strong dependence on LAT1, whereas the residual uptake of BPA under LAT1 inhibition suggests contributions from additional transporter systems (e.g., LAT2 and ATB^0+^), as previously reported [7].

The retention assays further underscore a key difference between BPA and 5F-αMe-3BPA at the cellular level. Under amino acid-free conditions, both compounds exhibited relatively slow efflux. However, in an amino acid-containing medium, BPA was rapidly extruded, whereas 5F-αMe-3BPA showed significantly slower efflux, maintaining a substantial intracellular fraction even in the presence of competing amino acids. This difference likely reflects differential susceptibility to LAT-mediated antiport exchange with extracellular amino acids. One plausible explanation is that 5F-αMe-3BPA is a poor substrate for LAT1 efflux activity or other amino acid exchangers that readily mediate BPA export, possibly due to the altered interaction profile conferred by α-methylation. However, this mechanism warrants further validation.

Collectively, these in vitro results establish that 5F-αMe-3BPA possesses three key features: (i) LAT1 affinity comparable to that of BPA, (ii) markedly higher LAT1/LAT2 selectivity than that of BPA, and (iii) superior intracellular retention in the presence of competing amino acids. High intracellular retention is particularly important for BNCT, since clinical BPA-BNCT protocols typically employ a 1-h neutron irradiation following a 2-h intravenous infusion [23]. 5F-αMe-3BPA, with high affinity, high selectivity, and prolonged intracellular retention, likely contributes to improved therapeutic efficacy and reduced normal tissue toxicity, positioning 5F-αMe-3BPA as a promising next-generation BNCT agent.

Based on the favorable cellular characteristics of 5F-αMe-3BPA, we hypothesized that its superior intracellular retention would translate into enhanced tumor boron accumulation in the late post-administration phase. We observed that although 5F-αMe-3BPA preferentially accumulated in tumors and exhibited favorable tumor-to-plasma and tumor-to-muscle ratios at 60 min, as previously reported [16,17], plasma boron concentrations declined rapidly, with the same tendency detected in the kidney, resulting in lower absolute tumor boron levels in the late phase than would be expected. Although previous studies that observed low plasma and high kidney boron levels suggested renal elimination of the compound [16,17], the parallel and rapid decline of plasma and kidney boron levels over time in this study strengthens the possibility that 5F-αMe-3BPA is efficiently excreted from the kidneys via altered transporter-mediated fluxes across basolateral and apical membranes that together facilitate rapid urinary excretion. Based on previous reports that halogenated and α-methylated aromatic amino acids can serve as substrates for OAT1-dependent tubular secretion [18,19], we hypothesized that OAT1 represents a major contributor to the rapid renal clearance of 5F-αMe-3BPA.

To test this hypothesis and improve the delivery of boron to tumors, we evaluated probenecid, a clinically used OAT inhibitor with oral bioavailability and a well-established safety profile [24,25,26]. Probenecid has been used clinically for decades to delay renal excretion and enhance the therapeutic efficacy of penicillin [27] and oseltamivir [28], providing a strong precedent for this pharmacokinetic strategy. Probenecid pretreatment successfully reduced early renal uptake, increased plasma boron exposure, and enhanced tumor accumulation. Importantly, probenecid did not compromise the intrinsic tumor selectivity of 5F-αMe-3BPA. Additionally, boron accumulation in nontarget tissues such as muscle, skin, brain, and bone remained low over time. Pancreatic accumulation, representative of LAT1-expressing normal tissues [22], was also increased by probenecid, confirming that the enhancement of boron accumulation reflects prolonged systemic exposure rather than altered LAT1 selectivity. Notably, the liver boron concentration was elevated in the probenecid group, likely reflecting redistribution to hepatobiliary clearance pathways when renal excretion is impaired. These results demonstrate that the pharmacological modulation of renal clearance is an effective strategy to increase tumor delivery of 5F-αMe-3BPA without compromising its intrinsic selectivity.

Increased retention by probenecid has been investigated for nuclear medicine imaging [29,30] and therapy [31] using α-methylated amino acids. However, to the best of our knowledge, this study was the first to explore probenecid as a means of enhancing tumor boron delivery for BNCT, opening a new avenue for pharmacokinetic optimization in this field. A unique advantage of this strategy for BNCT is that the therapeutic and side effects are confined to the neutron irradiation field, minimizing the risk of systemic toxicity from increased drug levels in tissues outside the irradiation field. This compatibility between probenecid-mediated pharmacokinetic enhancement and BNCT’s spatially confined cytotoxicity potentially represents a favorable safety profile. 5F-αMe-3BPA with probenecid pretreatment resulted in higher tumor boron levels for extended periods. Therefore, it may improve treatment outcomes, expand the range of treatable tumors, and enable dose reduction. In this study, we selected intraperitoneal administration of probenecid at 400 mg/kg, 60 min before 5F-αMe-3BPA injection, based on previous preclinical studies investigating pharmacokinetic modulation by probenecid [29]. Using body surface area conversion, 400 mg/kg in mice corresponds to approximately 32 mg/kg in humans [32], which is about 1.9 g for a 60-kg adult. This value is close to the upper end of clinically used doses of probenecid. At the same time, further optimization of the dose, route, and timing of probenecid administration will be necessary in future translational studies, taking interspecies differences into account.

This study had several limitations. First, tissue boron concentrations were determined using ICP-MS, and the interpretation of agent-derived boron assumes that the measured boron reflects intact compounds. While prior work has suggested that the compound can be excreted intact in urine after in vivo administration [17], the chemical form in tissues was not directly characterized in this study and should be evaluated in future translational studies. Second, direct evidence of OAT1-mediated transport of 5F-αMe-3BPA was not obtained using dedicated in vitro transporter expression systems. Thus, the contribution of other renal transporters to its clearance remains to be fully elucidated. Third, comprehensive pharmacokinetic analysis was limited by the necessity to sacrifice animals at discrete time points for biodistribution studies, precluding continuous assessment of plasma concentration-time profiles and calculation of rigorous pharmacokinetic parameters.

Recently, we developed 5-[^18^F]F-αMe-3BPA, the PET imaging counterpart of 5F-αMe-3BPA [17]. PET imaging with 5-[^18^F]F-αMe-3BPA might address several of these limitations. This structurally matched theranostic pair could enable noninvasive assessment of tumor LAT1 expression for patient stratification and quantitative evaluation of in vivo pharmacokinetics under probenecid pretreatment, enabling image-based analysis of the impact of probenecid on boron delivery. Imaging-guided BNCT could facilitate individualized treatment planning, optimization of drug dosing and timing relative to neutron irradiation, and early identification of patients most likely to benefit from 5F-αMe-3BPA-based BNCT. Therefore, integration of PET imaging into BNCT protocols represents a promising avenue toward precision medicine in this field.

## 5. Conclusions

This study quantitatively demonstrates that 5F-αMe-3BPA possesses LAT1 affinity comparable to that of BPA, markedly higher LAT1/LAT2 selectivity, and superior intracellular retention. Moreover, we identified probenecid pretreatment as a clinically translatable strategy to enhance tumor boron delivery by modulating renal clearance without compromising tumor selectivity. These findings establish a robust preclinical foundation for advancing 5F-αMe-3BPA toward clinical evaluation as a next-generation BNCT/PET theranostic agent and introduce pharmacokinetic optimization via OAT inhibition as a novel strategy to maximize therapeutic boron delivery in BNCT.

## Data Availability

The data supporting the results and findings of this study are available within the paper and the Supplementary Information files. Additional raw data are available from the corresponding author upon request.

## Supplementary Materials

The following supporting information can be downloaded at: https://www.mdpi.com/article/10.3390/pharmaceutics18050604/s1, Table S1: Effect of probenecid on the biodistribution of 5F-αMe-3BPA in T3M-4 tumor-bearing mice; Table S2: The area under the %ID/g–time curve (%ID/g·min, AUC) after administration of 5F-αMe-3BPA in T3M-4 tumor-bearing mice with or without probenecid pretreatment.
