# Supplementary material for: Preclinical Evaluation of 5F-αMe-3BPA for Improving Pharmacokinetics in Boron Neutron Capture Therapy
Source: Pharmaceutics. 2026 May 15;18(5):604. doi: 10.3390/pharmaceutics18050604 (PMC13211152; doi:10.3390/pharmaceutics18050604)
Supplement: Supplementary file 1 [file pharmaceutics-18-00604-s001.zip › pharmaceutics-4276914-supplementary.pdf]

Supplementary Information for:

Preclinical evaluation of 5F-αMe-3BPA for improving pharmacokinetics in Boron Neutron Capture Therapy

Naoya KONDO, Fuko HIRANO, Saki IRITANI,

Kensuke SUZUKI, Anna MIYAZAKI, Takashi TEMMA

Table S1. Effect of probenecid on the biodistribution of 5F-αMe-3BPA in T3M-4 tumor-bearing mice.

	10 min		60 min		180 min	
	**Ctrl (*n* = 6)**	**Probenecid (*n* = 5)**	**Ctrl (*n* = 10)**	**Probenecid (*n* = 5)**	**Ctrl (*n* = 6)**	**Probenecid (*n* = 5)**
**T3M-4**	6.9 ± 1.9 (*n* = 5)	6.5 ± 1.8 (*n* = 4)	6.2 ± 1.2	12 ± 2.7*** (*n* = 4)	3.7 ± 1.0 (*n* = 5)	5.9 ± 1.1* (*n* = 4)
**Plasma**	5.3 ± 1.2	9.1 ± 3.3*	0.8 ± 0.1	1.0 ± 0.3*	0.1 ± 0.0	0.2 ± 0.1*
**Muscle**	0.9 ± 0.2	1.6 ± 0.6*	0.3 ± 0.1	0.3 ± 0.1	0.2 ± 0.1	0.1 ± 0.0
**Skin**	3.5 ± 0.8	4.7 ± 1.5	1.1 ± 0.3	1.3 ± 0.3	0.3 ± 0.1	0.6 ± 0.1**
**Pancreas**	23 ± 7.1	23 ± 6.7	13 ± 3.8	29 ± 4.5***	1.9 ± 1.0	6.8 ± 3.6**
**Kidney**	46 ± 11	30 ± 6.0*	14 ± 3.8	17 ± 2.1	1.5 ± 0.7	2.2 ± 0.6
**Liver**	5.6 ± 1.2	5.0 ± 0.8	2.0 ± 0.5	5.0 ± 0.9***	0.2 ± 0.1	0.7 ± 0.2**
**Brain**	0.2 ± 0.0	0.2 ± 0.1	0.1 ± 0.0	0.1 ± 0.0	0.1 ± 0.0	0.1 ± 0.0
**Bone**	1.8 ± 0.6	2.1 ± 0.5	1.1 ± 0.8	0.9 ± 0.2	0.2 ± 0.3	0.4 ± 0.2
**Lung**	3.3 ± 0.8	7.0 ± 3.0*	1.2 ± 0.3	1.8 ± 0.5**	0.4 ± 0.1	0.6 ± 0.1*
**Heart**	1.3 ± 0.3	2.6 ± 1.0*	0.4 ± 0.2	0.6 ± 0.1*	0.3 ± 0.1	0.3 ± 0.1
**Spleen**	3.2 ± 0.6	4.2 ± 0.9*	1.5 ± 0.4	2.5 ± 0.7**	0.4 ± 0.1	0.6 ± 0.1*
**Tumor/Plasma**	1.2 ± 0.2	0.9 ± 0.3*	7.8 ± 1.9	14 ± 3.4*	49 ± 36	37 ± 9.7
**Tumor/Muscle**	7.8 ± 1.9	5.0 ± 2.1	22 ± 5.9	41 ± 10***	24 ± 8.4	45 ± 4.6**

Values are expressed as percentage of injected dose per gram of tissue (%ID/g, mean ± SD). Sample sizes are shown in parentheses where they differ from the group size indicated in the column header. *$p < 0.05$, **$p < 0.01$, ***$p < 0.001$ versus control at the same time point (unpaired t-test). The 60-min control data were reproduced from our previous study (Ref. 17)

Table S2. The area under the %ID/g–time curve (%ID/g·min, AUC) after administration of 5F-αMe-3BPA in T3M-4 tumor-bearing mice with or without probenecid pretreatment

	Ctrl			Probenecid			AUC ratio (Probenecid/Ctrl)		
	$AUC_{10\text{-}60}$	$AUC_{60\text{-}180}$	$AUC_{10\text{-}180}$	$AUC_{10\text{-}60}$	$AUC_{60\text{-}180}$	$AUC_{10\text{-}180}$	$AUC_{10\text{-}60}$	$AUC_{60\text{-}180}$	$AUC_{10\text{-}180}$
**T3M-4**	328	594	922	469	1088	1557	1.43	1.83	1.69
**Plasma**	152	54	205	254	72	326	1.67	1.34	1.59
**Muscle**	29	27	56	48	25	73	1.65	0.93	1.30
**Skin**	116	86	203	148	108	257	1.27	1.25	1.27
**Pancreas**	920	918	1838	1306	2139	3445	1.42	2.33	1.87
**Kidney**	1523	961	2484	1180	1170	2350	0.77	1.22	0.95
**Liver**	188	131	319	249	342	591	1.32	2.61	1.85
**Brain**	9	12	21	9	13	22	1.02	1.09	1.06
**Bone**	74	78	152	75	76	151	1.02	0.97	0.99
**Lung**	114	99	213	221	149	370	1.94	1.50	1.74
**Heart**	44	41	85	82	58	140	1.87	1.43	1.65
**Spleen**	118	115	233	170	190	359	1.44	1.65	1.54

AUC for each tissue was estimated by using linear trapezoidal method.

$AUC_{10\text{-}180} = AUC_{10\text{-}60} + AUC_{60\text{-}180}$

For AUC estimation, part of the 60-min control data were reproduced from our previous study[17].
